# Complete chloroplast genome sequence of *Pueraria lobata* (Willd.) Ohwi (Fabaceae): a traditional Chinese medicinal herb

**DOI:** 10.1080/23802359.2019.1694850

**Published:** 2019-12-09

**Authors:** Jiahui Sun, Ling Wang, Yuan Yuan, Rongrong Zhou, Haiyan Duan, Yuping Zhao

**Affiliations:** National Resource Center for Chinese Materia Medica, China Academy of Chinese Medical Sciences, Beijing, China

**Keywords:** Chloroplast genome, *Pueraria lobata*, genomic resources

## Abstract

*Pueraria lobata* is a traditional Chinese herb which has been used medically since ancient times. In this study, we sequenced the complete chloroplast genome of *P. lobata* based on next-generation sequencing and used the data to assess genomic resources. The chloroplast genome of *P. lobata* is 153,411 bp in length consisting of large and small single-copy regions of length 84,142 and 17,989 bp, separated by two IR regions of 25,640 bp. The overall GC content was 35.4%. *De novo* assembly and annotation showed the presence of 112 unique genes with 78 protein-coding genes, 30 tRNA genes, and four rRNA genes. A maximum-likelihood phylogenomic analysis showed that *Pueraria* was sister to *Pediomelum + Glycine*.

*Pueraria lobata* (Willd.) Ohwi is a traditional Chinese herb which has been used medically since ancient times. However, only a few genomic resources have been explored. Chloroplast genomes are valuable sources of genetic markers for phylogenetic analyses, genetic diversity evaluation, and plant molecular identification (Dong et al. 2012, 2016). Here, we sequenced and analyzed the chloroplast genome of *P. lobata* based on the next-generation sequencing method (Dong et al. 2013). The main goals of this study were to establish and characterize the organization of the whole chloroplast genome of *P. lobata* and to retrieve valuable genomic resources for this species.

We collected fresh healthy leaves from *P. lobata* species growing in the Anren, Hunan province. Voucher specimen and DNA sample were stored in the herbarium of the Institute of Chinese Materia Medica (CMMI), China Academy of Chinese Medical Sciences with accession number 431028LY1034. Total genomic DNA was extracted and purified following the method of Li et al. (2013). Paired-end (2 × 150 bp) sequencing was performed by Novogene Bioinformatics Technology Co. Ltd. (Beijing, China), using the Illumina Hiseq X-Ten platform. The paired-end reads were qualitatively assessed and assembled with SPAdes 3.6.1 (Bankevich et al. 2012). The annotation was performed with Plann (Huang and Cronk 2015). The annotated genomic sequence had been submitted to GenBank with the accession number MN180247.

The chloroplast genome of *P. lobata* is 153,411 bp in length consisting of large and small single-copy regions of length 84,142 and 17,989 bp, separated by two IR regions of 25,640 bp. GC content was 35.4%. The genome consisted of 112 different coding genes, including 78 protein-coding genes, 30 distinct tRNA genes, and four rRNA genes. Phylogenetic analyses were performed using maximum likelihood (ML) in RAxML 8.0 using the concatenated coding sequences of 77 chloroplast coding genes for a group of 23 species (Stamatakis 2014). Supports for nodes were calculated via rapid bootstrap analyses of 1000 replicates with GTR + G model. The reconstructed phylogeny revealed that *Pueraria* was sister to *Pediomelum*+*Glycine* (Figure 1). The whole chloroplast genome sequences provided sufficient genetic information for species identification and phylogenetic reconstruction of the genus *Pueraria*.

**Figure 1.**
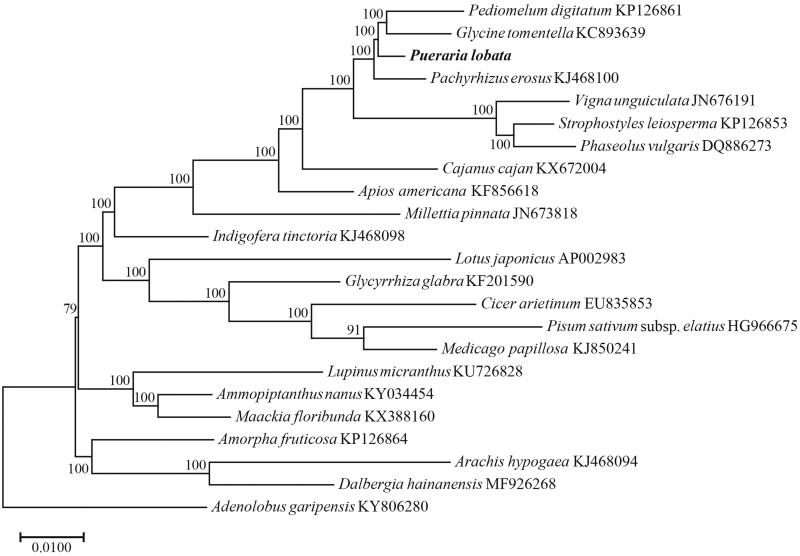
Phylogenetic tree reconstruction of 23 taxa using maximum likelihood (ML) methods based on 77 genes in the chloroplast genome sequences. ML bootstrap support value presented at each node.
